# Malignant pheochromocytoma: A diagnostic and therapeutic dilemma

**DOI:** 10.1016/j.ijscr.2021.106009

**Published:** 2021-05-24

**Authors:** Issam Jandou, Amine Moataz, Mohammed Dakir, Adil Debbagh, Rachid Aboutaieb

**Affiliations:** Ibn Rochd University Hospital Casablanca, Morocco; Faculty of Medicine and Pharmacy of Casablanca, Morocco

**Keywords:** Adrenal pheochromocytoma, malignant pheochromocytoma, Methoxylated derivative, Adrenalectomy, Metastasis, Menard triad

## Abstract

**Introduction:**

Malignant pheochromocytomas are rare endocrine tumors that develop within chromaffin tissue. The diagnosis of malignancy is based on neoplastic recurrence or the presence of metastasis in organs that lack chromaffin tissue. We report a series of four cases because of their diagnostic and therapeutic particularities.

**Presentation of case:**

we describe four clinical cases of patients with malignant pheochromocytoma whose Menard triad “headache-palpitations-sweating” was present in three out of four patients, the methoxylated derivatives were measured in 4 patients, 3 of which had high values, all of our patients carried out a CT scan which objectified signs of malignancy, MRI was performed on a single patient; presenting with a neoplastic recurrence; looking for a locoregional invasion.

**Discussion:**

Pheochromocytoma (PC) is a rare neuroendocrine tumor derived from the chromaffin cells of the adrenal medulla. Its annual incidence is 2 to 8 per million adults. A peak frequency is observed between 30 and 40 years of age. Approximately 10% of pheochromocytomas are malignant and in 10% of cases, bilateral localization is observed. Criteria for malignancy include the invasion of neighboring organs, a large tumor, the presence of lymphadenopathy on imaging, or fixation on scintigraphy. Surgery for MAP is not always curative. In the case of multiple liver metastases, treatment is based on adrenalectomy, which can be effectively combined with chemoembolization, cryoablation, or radiofrequency techniques.

**Conclusion:**

The main prognostic factors of the malignant pheochromocytomas are a large tumor volume, the existence or number of visceral metastases, and the presence of a mutation in the SDHB (Succinate dehydrogenase B) gene.

## Highlights

• Malignant pheochromocytomas are rare endocrine tumors that develop within chromaffin tissue.
• A very large part of pheochromocytomas are of genetic origin which can be part of hereditary syndromes.
• Criteria for malignancy include the invasion of neighboring organs, a large tumor, the presence of lymphadenopathy on imaging, or fixation on scintigraphy.
• Adequate management of this pathology requires a multidisciplinary consultation meeting

## 1 Background

Pheochromocytomas are rare adrenal tumors arising from chromaffin cells of the adrenal medulla. The prevalence of malignant pheochromocytomas is estimated at 10%, this figure can vary between 5 and 26%, the malignancy of the tumor is judged at first diagnosis or recurrence [1].

40% of pheochromocytomas are of genetic origin which can be part of hereditary syndromes (multiple endocrine neoplasia type2, neurofibromatosis type1, von-Hippel-Lindau disease…). These clinical presentations have been reported in accordance with PROCESS 2020 [2].

The objective of the treatment of these malignant tumors is to improve the quality and survival of patients by controlling catecholamine secretions and reducing tumor volume. Adequate management requires a multidisciplinary consultation meeting [3,4,5].

## 2 Case presentation

### 2.1 Case 1

Patient 1, aged 45 years old was operated in 2010 for right adrenal pheochromocytoma with a post-operative evolution without abnormality. 8 years later, the patient presented with headache and paroxysmal hypertension evolving in a context of altered general condition. Physical examination revealed a mass of the right hypochondrium of firm consistency.

The bioassessment had found normal serum adrenaline and dopamine levels, an elevation of noradrenaline. The urinary methoxylated derivatives metanephrine, and normetanephrine and Vanillylmandelic acid (VMA) were elevated.

**Fig. 1:**
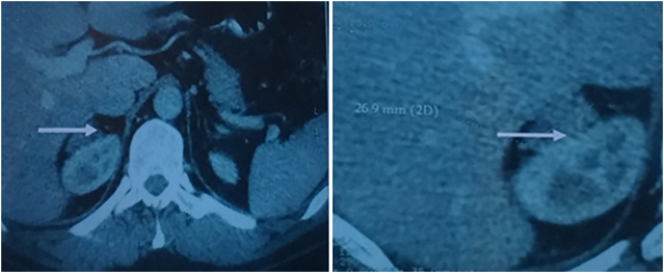
Abdominal CT shows the presence of a large heterogeneous tumor process, of tissue density.

AN abdominal CT scan had revealed a probable local recurrence with the presence of a voluminous heterogeneous tumor process, tissue density with a zone of necrosis and elevation after injection of contrast material, measuring 201/27 mm, occupying the hepato-renal space having intimate contact with the upper pole of the right kidney (Fig. 1). The patient underwent right adrenalectomy.

Histologic examination provided evidence of a pheochromocytoma greater than 10 cm in size and weighing approximately 200 mg, necrotic reshaping, capsular invasion with evidence of malignancy.

The postoperative course was satisfactory over an 18-month follow-up, the disappearance of clinical signs and the normalization of methoxylated derivatives.

### 2.2 Case 2

Patient 2, 44 years old, has had paroxysmal hypertension for two years with pulsating headache, palpitations, lipothymia, and night sweats. Physical examination showed hypertension at 14/8 mmHg under antihypertensive treatment and a mass sensitivity to palpation of the lumbar fossa.

The patient underwent an abdominal CT scan which revealed a 6/7 cm right adrenal mass of tissue density and cystic fibrosis. The laboratory workup showed an elevation of the urinary **VMA**. The patient benefited from a right adrenalectomy, the pathological examination of the surgical specimen was in favor of a pheochromocytoma.

6 months later, the patient presented abdominal pain with no palpable mass, ultrasound examination revealed two hepatic nodules, and the methoxylated derivatives dosage was normal. Hepatic MRI had visualized two hepatic nodules in hypo signal T1 and hyper signal T2 at segments 3 and 4 measuring 1.5 and 2 cm in diameter respectively. These two lesions rise intensely and transiently at arterial time and become almost isointense at hepatic parenchyma at late time. MIBG scintigraphy revealed two fairly intense hyperfixation foci in the liver.

The patient underwent a laparotomy by the team of visceral surgeons who ensured the establishment of two hepatic nodules. The pathology study was in favor of a hepatic metastasis of a pheochromocytoma.

The postoperative course was favorable during a 15-month follow-up.

### 2.3 Case 3

Patient 3, 50 years old, diabetic on insulin, with a history of a total thyroidectomy 8 years ago (Papillary Carcinoma). Admitted for the management of two adrenal masses revealed by Menard's triad of headaches, palpitations, and sweating with severe hypertension resistant to triple therapy evolving for 5 months.

Thoracic-operative CT scan had shown two bilateral voluminous adrenal masses. That measuring 4/3 cm on the right and 13/11 cm on the left, hypodense, heterogeneous after injection of PDC. The left mass was the site of central necrosis and calcifications that displaced the kidney and the spleen, celiacomesenteric adenopathies measuring 5/6 cm which displaced the ICV and a left pulmonary parenchymatous nodule measuring 13 mm, Liver full hypodense nodules which were raised after injection of PDC. The bioassessment showed an increase in methoxylated drifts.

After the discovery of the adrenal masses, a strong suspicion of multiple endocrine neoplasia exegeted a rereading of the thyroidectomy slides which objectified a medullary carcinoma of the thyroid.

A NEM2 (Multiple endocrine neoplasia 2) assessment was performed, cervical ultrasound exploration was its evolutionary anomaly - Calcitonin assay 31 ng/l. The hyperparathyroidism test shows a blood calcium level of 99 mg/l, PTH 5.64 (normal 1.07–7.74), low VIT D. A bone scan did not show any evidence of secondary localization.

The patient was transferred to the urology department for tumor size reduction by laparotomy after a multidisciplinary consultation meeting. A pre-anesthetic consultation as well as a pre-operative preparation was carried out. The immediate postoperative course was unremarkable. The postoperative course was marked by the death of the patient 5 months after surgery.

### 2.4 Case 4

Patient 4, 31 years old, with a history of left adrenalectomy for pheochromocytoma. The evolution was marked by hypertensive peaks associated with an increase in methoxylated derivatives. The abdominal computed tomography showed multiple hypodense left retroperitoneal lesions taking heterogeneous PDC measuring 45/20 mm, from the adrenal compartment to the lower pole of the kidney. MIBG scans showed asymmetry in sub-renal absorption.

The evaluation of the clinical condition of the patient contraindicates the operation. Palliative chemotherapy has been indicated, based on cyclophosphamide, vincristine and dacarbazine.

**Fig. 2:**
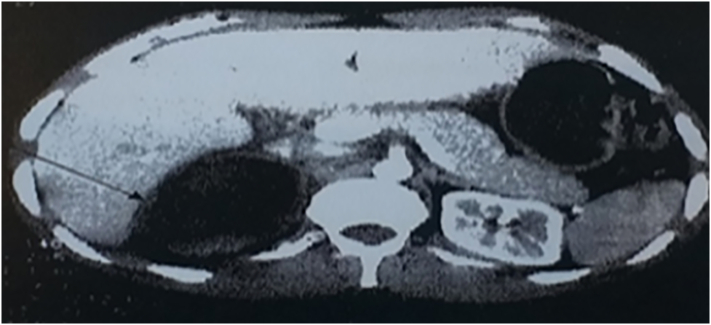
abdominal CT scan which revealed a 6/7 cm right adrenal mass of tissue density and cystic fibrosis.

**Fig. 3:**
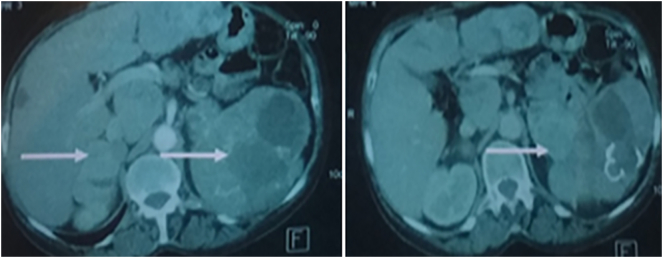
CT scan had shown two bilateral voluminous adrenal masses measuring 4/3 cm on the right and 13/11 cm on the left, hypodense, heterogeneous after injection of PDC.

**Fig. 4:**
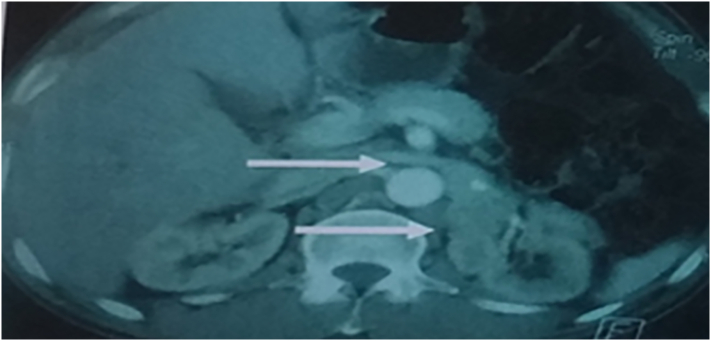
CT showed multiple hypodense left retroperitoneal lesions taking PDC heterogeneously measuring 45/20 mm, from the adrenal lodge to the lower pole of the kidney.

The patient's follow-up was marked by the death of the patient after 5 months of his first chemotherapy treatment. (See Fig. 2, Fig. 3, Fig. 4.)

## 3 Discussion

Pheochromocytoma (PC) is a rare neuroendocrine tumor derived from the chromaffin cells of the adrenal medulla. Its annual incidence is 2 to 8 per million adults. A peak frequency is observed between 30 and 40 years of age. Approximately 10% of pheochromocytomas are malignant and in 10% of cases, bilateral localization is observed [5]. A mutation can be found in 25% of cases, even in patients with an “apparently” sporadic pheochromocytoma [6].

Criteria for malignancy include the invasion of neighboring organs, a large tumor, the presence of lymphadenopathy on imaging, or fixation on scintigraphy.

The traditional clinical presentation is variable arterial hypertension accompanied by paroxysmal vasomotor disorders of which the Menard triad, headache-palpitation-sweat is the most classic manifestation [7]. This counterpart is not always complete, in our series, this triad was found in 3 of the 4 patients, 2 of whom do not have a complete symptomatology.

High blood pressure is the cardinal sign of pheochromocytoma, the permanent character is the most frequent clinical form, severe, systolic-diastolic, extremely unstable, and refractory to treatment [8].

The paroxysmal hypertension is the most evocative form, the paroxysmal crisis begins abruptly with ascending diffuse pain such as cramps in the upper limbs associated with intense headaches, anxiety, deep sweating with a rise in blood pressure that can be complicated by a serious acute accident, Acute pulmonary edema, Acute Myocardial Infarction or transient blindness [9].

In our series, four patients had paroxysmal hypertension, and only one in four patients had permanent hypertension.

Plasma catecholamine dosing is no longer performed as a first-line treatment, reserved for patients with hypertension at the time of collection, thus eliminating the risk of a false negative. Moreover, this dosage still has prognostic value [9].

The metanephrine (MN) assays are performed as a first-line procedure and are based either on the assay of free plasma metanephrines and/or the assay of fractionated plasma or urinary metanephrines [10].

Plasma free metanephrines assess the direct secretion produced by the tumor continuously as opposed to the episodic secretion of catecholamines, which reflect the tumor mass. Their measurement would be little affected by sympathoadrenal excitation. Free metanephrines are rapidly cleared from the circulation by an extraneuronal uptake mechanism. They are therefore little affected by renal failure [11].

Stress and physical effort can also be responsible for an increase in urinary free adrenaline and norepinephrine, their major interest lies in the paroxysmal forms, a dosage 3 h after the onset of the crisis [12].

Urinary fractional metanephrines are measured after deconjugation. The forms measured are free and conjugated MN. Conjugated MN is slowly eliminated by the kidney, and by the liver. Therefore, they are more likely to be disturbed by kidney failure, and by the contribution of the gastrointestinal tract [13]. The vanyl-mandelic acid (VMA) assay is not a good marker for the diagnosis of pheochromocytoma [8].

In our series, plasma catecholamine dosing was performed in a single patient. Also, three of our patients were tested for methoxylated derivatives and VMA.

Abdominopelvic computed tomography (CT) scan with and without contrast agent injection is currently the reference method.

It has a high sensitivity (98%) and can detect adrenal tumors as small as 0.5 cm [14]. The radiological signs in favor of pheochromocytoma malignancy are [15]:

■ The size: the tumor size as an argument for malignancy is respectively by 20%, 65% and 89% for diameters ≥4 cm, ≥6 cm, and ≥8 cm
■ The irregular contours, the heterogeneity.
■ Venous or contiguous invasion
■ The presence of lymphadenopathies
■ A density greater than 20 HU
■ Characteristic slow washout (> or <10–15 min)
■ The presence of metastasis.

In our series, all patients were given a CT scan with criteria for malignancy were: large size; the presence of metastases nodes and remote lymph nodes, and locoregional invasion.

MRI is considered by many authors to be superior to CT in the diagnosis of pheochromocytoma since it allows a better assessment of local invasion and possible invasion of the inferior vena cava than CT [16].

In our series, MRI was performed on a single patient; presenting with a neoplastic recurrence; looking for a locoregional invasion.

The 5-year survival of PCM is estimated at 40 to 77%. The increase in tumor mass is the main cause of death in PMKs. Tumor control must, therefore, be the main objective of the management of PMKs. However, clinical manifestations due to excess catecholamines (high blood pressure…) must be treated, as they are thought to be responsible for 30% of deaths due to Malign adrenal pheochromocytoma (MAP) [17].

The main prognostic factors are a large tumor volume, the existence or number of visceral metastases, and the presence of a mutation in the SDHB (Succinate dehydrogenase B) gene [18].

Treatment is usually multimodal. It is based on excisional surgery (adrenalectomy at the healthy margin and excision of metastases), pharmacological (drug) control of hormonal symptoms, and systemic treatments.

Preoperative pharmacological preparation aims to prevent the deleterious consequences of the inevitable catecholamine discharges during surgery. The reference treatment is based on alpha-blockers, administered 7 to 14 days before the operation [19].

Surgery for MAP is not always curative. In the case of multiple liver metastases, treatment is based on adrenalectomy, which can be effectively combined with chemoembolization, cryoablation, or radiofrequency techniques.

In cases of metastatic or unresectable MAP, surgical debulking may be discussed for symptomatic purposes, although it would not improve survival [20].

Chemotherapy may be offered to treat metastatic or recurrent malignant pheochromocytoma. The most common combination of chemotherapy drugs used is the CVD protocol, which includes cyclophosphamide (Procytox), vincristine (Oncovin) and dacarbazine (DTIC) [19].

Active monitoring or delayed treatment may be indicated for inoperable patients with a median survival greater than 10 years. This monitoring is based on clinical and radiological examination (every 3 months then 6 months then annually). Three arguments prohibit active surveillance, including hormonal disturbance, high tumor volume (≥7 bone metastases, involvement hepatic ≥50% of parenchyma, multiple pulmonary nodules larger than 2 cm), or radiological progression [18].

## 4 Conclusion

Malignant pheochromocytoma is rare, the diagnosis of malignancy may be made preoperatively or immediately postoperatively, but sometimes it is made several years after the initial diagnosis, hence the need for life-long monitoring of patients operated on for pheochromocytoma.

## Abbreviations

PC: pheochromocytoma
MAP: malign adrenal pheochromocytoma
NEM2: (multiple endocrine neoplasia 2)
MN: metanephrine
CT: computed tomography
MIBG: métaiodobenzylguanidine
VMA: vanillylmandelic acid

## Provenance and peer review

Not commissioned, externally peer-reviewed.

## Declarations

Ethics and consent to participate to the Declarations: Not applicable.

## Consent to publication

The consent to publish this information was obtained from study participants. We confirm that the proof of consent to publish from study participants are available when requested and at any time.

## Availability of data and material

The datasets in this article are available in the repository of the urology database, CHU Ibn Rochd Casablanca, upon request, from the corresponding author.

## Funding

Not applicable.

## Author's contributions

Dr. IJ, Dr. AM, Dr. YL, Dr. GM analysed and performed the literature research, Pr. MD, Pr. AD, Pr. RA performed the examination and performed the scientific validation of the manuscript. IJ was the major contributors to the writing of the manuscript. All authors read and approved the manuscript.

## Declaration of competing interest

The authors state that they do not have competing interests.
